# Long Non-Coding RNAs in the Pathogenesis of Diabetic Kidney Disease

**DOI:** 10.3389/fcell.2022.845371

**Published:** 2022-04-20

**Authors:** Mengsi Hu, Qiqi Ma, Bing Liu, Qianhui Wang, Tingwei Zhang, Tongtong Huang, Zhimei Lv

**Affiliations:** ^1^ Department of Nephrology, Shandong Provincial Hospital Affiliated to Shandong First Medical University, Jinan, China; ^2^ Department of Nephrology, Shandong Provincial Hospital, Cheeloo College of Medicine, Shandong University, Jinan, China

**Keywords:** long non-coding RNA, diabetic kidney disease, mesangial cell, glomerular endothelial cell, podocyte, tubular epithelial cell, pathogenesis

## Abstract

Diabetic kidney disease (DKD) is one of the major microvascular complications of diabetes mellitus, with relatively high morbidity and mortality globally but still in short therapeutic options. Over the decades, a large body of data has demonstrated that oxidative stress, inflammatory responses, and hemodynamic disorders might exert critical influence in the initiation and development of DKD, whereas the delicate pathogenesis of DKD remains profoundly elusive. Recently, long non-coding RNAs (lncRNAs), extensively studied in the field of cancer, are attracting increasing attentions on the development of diabetes mellitus and its complications including DKD, diabetic retinopathy, and diabetic cardiomyopathy. In this review, we chiefly focused on abnormal expression and function of lncRNAs in major resident cells (mesangial cell, endothelial cell, podocyte, and tubular epithelial cell) in the kidney, summarized the critical roles of lncRNAs in the pathogenesis of DKD, and elaborated their potential therapeutic significance, in order to advance our knowledge in this field, which might help in future research and clinical treatment for the disease.

## Introduction

Diabetic kidney disease (DKD) is one of the major microvascular complications of diabetes mellitus, usually developed in patients who fail to control blood glucose at a stable and standard level over a long period of time. Epidemics showed that among hospitalized patients in China, the proportion of chronic kidney disease brought on by DKD has reached 0.71%, which has become a dominant reason over glomerulonephritis since 2011, when the percentage of the latter only accounted for 0.66% (Zhang et al., 2016). Globally, around 20% of 400 million diabetic patients were suffering from DKD (McGrath and Edi, 2019); consequently, there is an urgent necessity to develop a new diagnosis and therapeutic strategy to postpone the progression of diabetes mellitus and its complications.

DKD was traditionally regarded as a simple renaming of diabetic nephropathy (DN) (Anders et al., 2018), which, however, was not accurate enough for those who were clinically diagnosed with DKD but with pathological changes primarily characterized by other glomerular diseases including membranoproliferative glomerulonephritis and renal amyloidosis (Qi et al., 2017). In addition, whether proteinuria should be considered as a specific clinical indicator of renal complications in diabetic patients remained puzzling (Chen et al., 2017; Selby and Taal, 2020). This inconsistency existed in clinical manifestations, and pathological changes have seriously affected our understanding on DN. DKD seems to be a more comprehensive clinical description, including DN confirmed by renal biopsy and clinical diagnosis according to diabetes duration and relevant clinical indicators such as urine protein without pathological examination. Generally speaking, DKD links to oxidative stress, inflammatory responses, hemodynamic disorders *etc*. secondary to hyperglycemia (Selby and Taal, 2020), with renal tissue changes including mesangial expansion, glomerular basement membrane (GBM) thickening, nodular and global glomerulosclerosis, and lesions of tubulointerstitial and renal vessels (Tervaert et al., 2010). Until now, researchers have done extensive exploration on the pathogenesis of DKD, the etiology of which, however, remains largely indeterminate.

Long non-coding RNAs (lncRNAs), defined as transcripts longer than 200 nucleotides and with no capacity to encode proteins (Quinn and Chang, 2016), exist in eukaryotes and are transcribed by RNA polymerase II. LncRNAs, which were once seen as transcriptional noise (Wang et al., 2004; Struhl, 2007), have been demonstrated to be participating in many human diseases, such as tumor (Bhan et al., 2017; Flippot et al., 2019), cardiovascular diseases (Uchida and Dimmeler, 2015; Poller et al., 2018), neurodegenerative diseases (Riva et al., 2016; Wu and Kuo, 2020), and metabolic-related diseases (Sun and Lin, 2019). Typically, lncRNAs exerted the function of competitive endogenous RNA (ceRNA) by competitively binding miRNAs and regulating gene expression and various physiological processes (Beermann et al., 2016) at the transcriptional and posttranscriptional levels (Sun et al., 2018b). Amounts of research indicated that lncRNAs might also be involved in cell proliferation, differentiation, and apoptosis other than their roles in transcriptional regulation, meanwhile, manifested potential therapeutic values for DKD (Kato, 2018). In this review, we focused on abnormal expression and functions of lncRNAs in major resident cells of the kidney, summarized the critical role of lncRNAs in the pathogenesis of DKD, and elaborated their potential therapeutic significance.

## Long Non-Coding RNA and Glomerular Mesangial Cell Injury

Physiologically, glomerular mesangial cells (MCs) participate in the synthesis and degradation of extracellular matrix components and regulate the permeability of the glomerular filtration membrane (Abboud, 2012), while MCs similarly act as the principal targets in some immune-related glomerular diseases such as lupus nephritis and IgA nephropathy (Du et al., 2005; Suzuki et al., 2011) or metabolic diseases (Tung et al., 2018) including DKD. It is worth noting that the pivotal role of lncRNAs has been gradually highlighted in recent years in diabetic mesangial cell injury (Sui et al., 2012).

### Long Non-Coding RNA and Inflammation in Glomerular Mesangial Cell

Different from previous views that DKD was a metabolic-related disease irrelevant to inflammation, inflammatory mediators, and signaling pathways in the pathogenesis of DKD has recently gained wider acceptance for researchers (Galkina and Ley, 2006; Wada and Makino, 2013; Jung and Moon, 2021). CircLRP6 participated in HG-induced MC injury by upregulating the expression of Toll-like receptor 4 (TLR4), extensively involved in lipopolysaccharide-induced inflammatory cell injury (Chen et al., 2019a; Ciesielska et al., 2021). Notably, TLR4 was indicated to be a downstream target gene of early growth response-1 (EGR-1) (Ha et al., 2014; Wu et al., 2019), ectopic expression of which mediated the inflammatory process in various cell types including MCs (Li et al., 2017b; Shi et al., 2021). Experiments indicated valsartan could reduce TLR4 levels and decrease the secretion of TNF-α, IL-6 and IL-1β in STZ-induced diabetic mice *via* inhibiting EGR-1 expression (Ha et al., 2014).

Recent findings reported that lncRNAs exerted an indispensable effect in the inflammation of MCs under high glucose (HG) conditions. Reportedly, lncRNA maternally expressed gene 3 (MEG3), mainly expressed in the cytoplasm of MCs, could activate the EGR-1/TLR4 pathway by acting as an endogenous sponge of miR-181a, and further resulted in upregulated levels of inflammatory cytokines in the renal cortex of DN rat models, including CRP, IL-1β, IL-6, and MCP-1, which was also observed in HG-induced MCs (Zha et al., 2019). In addition, lncRNA ribonuclease P RNA component H1 (RPPH1) was involved in the inflammation of MCs by directly binding galectin-3 (gal-3), and subsequently activating the mitogen-activated protein kinase/extracellular signal-regulated kinase (MEK/ERK) pathway to promote the expression of MCP-1 and TNF-α, which, similarly, was detected in the renal cortex of DN mice and the cytoplasm of HG-induced MCs (Zhang et al., 2019b). Interestingly, it has been demonstrated that activation of the MEK/ERK signaling pathway could regulate the expression of EGR-1 (Mishra et al., 2006). However, whether activation of the MEK/ERK signaling pathway participated in the regulation of the EGR-1/TLR4 pathway in inflammatory response of HG-induced MCs needed further investigations.

Tissue inhibitors of metalloproteinases 3 (TIMP3) is a matrix metalloproteinase (MMP) inhibitor, differentially expressed in diabetic glomeruli (Woroniecka et al., 2011). In diabetic mice with TIMP3 deficiency, urinary albumin excretion was significantly increased, accompanied by elevation of MCP-1 and an increase of pro-fibrotic markers including pro-collagen type I-α1 and TGF-β (Basu et al., 2012). Other literatures reported that abnormal expression of TIMP3 was associated with renal inflammatory responses and fibrosis by activating various signaling pathways, including the Jun N-terminal Kinase (JNK) signaling pathway (Kassiri et al., 2009). In db/db diabetic mice, deterioration of urinary protein excretion was observed when phosphorylation levels of JNK were upregulated (Ijaz et al., 2009). Very recently, Zhu et al. (2021) showed that lncRNA cancer susceptibility candidate 2 (CASC2) was markedly decreased in HG-cultured MCs and was involved in the regulation of TIMP3 expression by sponging miR-135a-5p in order to inactivate JNK signaling transduction, which subsequently weakened the secretion of HG-induced MCP-1, TNF-α, and IL-6 and reduced the expression of TGF-β1, fibronectin (FN), and collagen-IV (col-IV).

Previous study has shown that ginsenoside Rg1 participated in LPS-induced cardiomyocyte inflammation by regulating the NF-κB pathway, which activated NOD-like receptor 3 (NLRP3) and stimulated macrophages to elevate the levels of IL-1β, IL-18, and TNF-α (Luo et al., 2020). Similarly, it was found that lncRNA Gm4419, highly expressed in the cytoplasm of MCs in HG and kidney tissue of DN mice, could directly interact with P50 (the subunit of NF-κB) to activate the NF-κB pathway, and further increased the expression of NLRP3 inflammasome, along with increased expression of pro-inflammatory cytokines including MCP-1, IL-1β, and TNF-α and renal fibrosis-related proteins including FN and col-IV (Yi et al., 2017). It seemed that a variety of lncRNAs were involved in inflammatory responses of MCs through their interplay with various inflammation-related signaling pathways, either by sponging multiple miRNAs or interacting with specific cytoplasmic proteins as critical signal mediators.

### Long Non-Coding RNA and Proliferation in Glomerular Mesangial Cell

Excessive proliferation of MCs was a significant pathological feature of various glomerular diseases, including lupus nephritis, IgA nephropathy, and DKD (Suzuki et al., 2011; Lei et al., 2019; Gao et al., 2020), with an increased synthesis of extracellular matrix protein such as col-IV, collagen-V (col-V), laminin (LN), and FN (Mason and Wahab, 2003), eventually promoting glomerular fibrosis of DKD (Chen et al., 2003). Until recently, Huang et al. (2019) found that lncRNA nuclear enriched abundant transcript 1 (NEAT1) was significantly upregulated in STZ-induced diabetic rats, with an elevation of clinical indexes such as urine protein, blood urea nitrogen (BUN), and creatinine, which was associated with activation of the AKT/mTOR signaling pathway, whereas knockdown of NEAT1 was able to inhibit proliferation of MCs and decrease the levels of extracellular matrix proteins including TGF-β1, FN, and col-IV. Significantly, mTOR was an important molecule that regulated autophagy function of podocytes (Boya et al., 2013), while, it is unclear whether NEAT1 led to cell proliferation by affecting the autophagy of MCs.

Wilms’ tumor protein 1 (WT1) was a zinc-finger like transcription factor and previously indicated to participate in the occurrence of renal tumors (Rivera and Haber, 2005), generally expressed in mature podocytes. Beyond that, studies have shown that WT1 was upregulated in urine of diabetic patients with heavy proteinuria and the levels of which were negatively correlated with renal function (Kalani et al., 2013). Fascinatingly, Zhong et al. (2020) reported that, lncRNA plasmacytoma variant translocation 1 (PVT1) was highly expressed in serum of DN patients and HG-induced human MCs, overexpression of which could upregulate WT1 expression by acting as a molecular sponge of miR-23b-3p and subsequently activated NF-κB signal transduction, ultimately promoted proliferation in HG-induced human MCs. Targeting PVT1 was able to inhibit MC proliferation and extracellular matrix protein deposition (Zhang et al., 2017), which seemed to be an interesting and novel perspective for delaying the progression of diabetic mellitus and the occurrence of renal complications.

Another lncRNA Dlx6os1 was found to be highly expressed in the kidney of DN mice and in the nuclei of HG-treated SV40 MES13 cells (Chen et al., 2022). Under HG conditions, high levels of Dlx6os1 accelerated the proliferation of HG-induced SV40 MES13 cells by recruiting the enhancer of zeste homolog 2 (EZH2), a key subunit of polycomb repressive complex 2 (PRC2), to SRY-related high-mobility group box 6 (SOX6) promoter to inhibit the expression of SOX6, accompanied by the elevation of TNF-α, IL-1β, and IL-6 and fibrosis-related proteins including col-IV, FN, and TGF-β1, while knockdown of Dlx6os1 expression showed a reversal effect, manifested as reduction of these inflammatory cytokines and fibrosis-related proteins (Chen et al., 2022). PRC2 is a methyltransferase catalyzing associated with chromatin regulation, with one of the core catalytic subunit EZH2 (56, 57). EZH2 could regulate the transcriptional activity of targeted genes by trimethylation of Lys-27 in histone 3 (H3K27me3) (Laugesen et al., 2019; Duan et al., 2020). SOX6 belongs to the SOX family, which is a transcription factor of the DNA-binding domain and participate in the regulation of target genes by binding with them in the promoter region (Kiselak et al., 2010). SOX6 has been reported to inhibit pancreatic β cell proliferation to negatively regulate insulin secretion (Iguchi et al., 2007) and participate in proliferation of renal tumor cells (Chen et al., 2020).

Vigilantly, abnormal increase of high-mobility group protein 2 (HMGA2) was detected in peripheral blood of DN patients (Alkayyali et al., 2013). HMGA2 was generally expressed in human embryonic stem cells and gradually decreased with the completion of development, while re-expressed in malignant proliferation of tumor cells, functioning as a pivotal regulatory factor (Mansoori et al., 2021). *In vitro* studies showed that upregulation of HMGA2 was triggered by lncRNA cyclin-dependent kinase inhibitor 2B antisense RNA 1 (CDKN2B-AS1) *via* sponging miR-424-5p after HG stimulation and resulted in proliferation of MCs and accumulation of the extracellular matrix (ECM) (Li et al., 2020b). Compared with the HG group, knockdown of CDKN2B-AS1 inhibited the expression of HMGA2 and significantly alleviated HG-induced proliferation of MCs and excessive secretion of extracellular matrix protein (Li et al., 2020b).

### Long Non-Coding RNA and Autophagy in Glomerular Mesangial Cell

Autophagy refers to the lysosomal protein degradation process associated with a series of proteins, such as ATGs, Beclin-1, and light chain 3 (LC3) and regulated by many signaling pathway proteins including mTOR, AMP activated protein kinase (AMPK), and silent information regulator T1 (SIRT1) (Lee et al., 2008; Boya et al., 2013; Lenoir et al., 2015). Specifically, the ROS-mediated ERK signaling pathway promoted the expression of Beclin-1 and LC3-II and triggered mitochondrial autophagy in MCs (Xu et al., 2016), which was indicated to be playing a partially protective role under HG conditions. It is worth noting that the differential expression of lncRNA has been identified in the occurrence of autophagy in MCs stimulated by HG treatment. The overexpression of lncRNA SOX2-overlapping transcript (SOX2OT) could significantly inhibit phosphorylation of AKT and mTOR in HG-induced MCs, causing autophagy of these MCs and alleviated renal pathological damage in STZ-induced diabetic mice (Chen et al., 2021). Of interest, the literature reported that SOX2OT also participated in podocyte autophagy by acting as a sponge of miR-9 (Zhang et al., 2019c). Nevertheless, it was still indefinite that whether the involvement of SOX2OT in MC autophagy was also in a miR-9/SIRT1-dependent manner and if there was a cross talk signaling between MC and podocyte autophagy.

## Long Non-Coding RNA and Glomerular Endothelial Cell Injury

Glomerular endothelial cells (ECs) are important components of glomerular filtration barrier (GFB), the fenestra on the surface of which could effectively prevent some tangible components in the blood from directly contacting with GBM (Fu et al., 2015). What is worthy of attention is that ECs are the dominant cells easily impaired by HG stimulation, and damage of which may further mediate the occurrence and development of diabetic vascular complications including cardiomyopathy and renal injury (Vulesevic et al., 2016; Lassén and Daehn, 2020). Multiple evidence have shown lncRNAs were differentially expressed in cardiovascular and renal endothelial cells (Puthanveetil et al., 2015; Huang et al., 2021), showing a close relationship between dysfunction of lncRNAs and diabetic endothelial injury or DKD.

### Long Non-Coding RNA and Inflammation in Glomerular Endothelial Cell

Research has found that long-term and mild inflammatory responses were associated with the dysfunction of ECs in db/db mice (Zhao et al., 2020). Serum amyloid A (SAA), a pro-inflammatory protein, was elevated in diabetic mice and in serum of diabetic patients (Anderberg et al., 2015; Wilson et al., 2018), the deposition of which in atheroma has been reported to cause endothelial dysfunction (Witting et al., 2011). LncRNA metastasis-associated lung adenocarcinoma transcript 1 (MALAT1), first described as a key prediction index in metastasis of lung cancer (Ji et al., 2003), was found to be critical in various diabetic complications including cerebrovascular disease and DKD (Abdulle et al., 2019). Recently, it was found that MALAT1 was overexpressed in human umbilical vein endothelial cells and STZ-induced diabetic mice, which could activate SAA3 to mediate EC injury by promoting the release of inflammatory cytokines such as IL-6 and TNF-α. The blockade of MALAT1 expression, on the contrary, inhibited aberrant secretion of these inflammatory molecules (Puthanveetil et al., 2015).

### Long Non-Coding RNA and Apoptosis in Glomerular Endothelial Cell

Apoptosis refers to pro-inflammatory and programmed cell death regulated by genes such as the caspase family in order to maintain the homeostasis of the internal environment (Elmore, 2007), which is determined by the equilibrium between antiapoptotic proteins such as Bcl-2 and pro-apoptotic protein such as Bax (Salam et al., 2018). Multiple studies have indicated that HG could induce apoptosis in ECs by upregulating pro-apoptotic protein and inhibited antiapoptotic protein levels (Su et al., 2018), an important step triggering endothelial injury that would initiate the development of diabetes mellitus and DKD (Dou and Jourde-Chiche, 2019). Reportedly, lncRNA KCNQ1-overlapping transcript 1 (KCNQ1OT1) was involved in the development of diabetic retinopathy (DR) by promoting proliferation and angiogenesis of hRECs through sponging miR-1470; and knockdown of KCNQ1OT1 induced apoptosis and inhibited cell proliferation in HG (Shao et al., 2019). Likewise, Jie et al. (2020) found that lncRNA KCNQ1OT1 was highly expressed in serum of DN patients, suggesting a critical involvement of lncRNA KCNQ1OT1 dysregulation in the pathogenesis of DKD, and in HG-cultured glomerular ECs, it was showed that lncRNA KCNQ1OT1 was overexpressed, with concomitant upregulation of sorbin and SH3 domain-containing protein 2 (SORBS2) by targeting miR-18b-5p, knockdown of KCNQ1OT1 increased the level of apoptosis rate detected by flow cytometry assay (Jie et al., 2020), suggesting a contradict instead of a unilaterally protective role by targeting KCNQ1OT1 in HG-induced endothelial cell injury. Further evidence were needed to verify its role in diabetic complications such as DKD. Also, the specific apoptotic proteins involved were not determined in the study.

### Long Non-Coding RNA and Endothelial Mesenchymal Transformation

Endothelial mesenchymal transformation (EndMT), a complex transversion of cell differentiation, which is an important source of fibroblasts, with the missing endothelial markers such as CD31 and the expression of mesenchymal proteins including α-SMA and FSP-1 (Srivastava et al., 2019). Studies have demonstrated that TGF-β/SMAD mediated EndMT could contribute to early renal fibrosis in diabetic mice (Li et al., 2009; Srivastava et al., 2021), and glomerulus EndMT could also induce epithelial mesenchymal transformation (EMT) of renal tubular epithelial cells (TECs) (Srivastava et al., 2021), which might work together to accelerate the process of renal fibrosis in DKD (Li et al., 2020a). Very recently, Shi et al. (2020) found that lncRNA H19 levels were increased in a time-dependent manner in human dermal microvascular endothelial cells with TGF-β2 treatment and renal tissue of STZ-induced CD1 mice, and that gene knockdown of H19 in diabetic mice partially restored renal function along with mitigated renal fibrosis, and increased expression of CD31 but decreased expression of FSP-1 in renal tissues of these gene-modified diabetic mice through inhibiting TGF-β/SMAD3 signaling transduction (Shi et al., 2020).

## Long Non-Coding RNA and Podocyte Injury

Podocytes are composed of cell bodies and foot processes (FPs) which cover the surface of GBM and connect with GBM through adhesion molecules such as α3β1 integrins and proteoglycan molecules such as heparan sulfate proteoglycans (Abrahamson, 2012). The FPs between adjacent podocytes cross each other to form a hiatal membrane called slit diaphragm (SD), which act as a critical barrier to prevent protein leakage with key component proteins including nephrin and podocin (Garg, 2018). Additionally, cytoskeleton proteins, including actin, synaptopodin, and other structural proteins, are of great significance to maintain the normal structure and function of podocytes (Garg, 2018). Reduction of podocyte density and abnormality of podocyte structure or function for any reason have been recognized to be of great significance to the occurrence and development of proteinuria. The following is a main introduction to the effects of lncRNAs on diabetic and HG-related podocyte injury.

### Long Non-Coding RNA and Inflammation in Podocyte

Research has found that TLRs existed as a conserved family of pattern recognition receptors in the innate immune system by increasing pro-inflammatory cytokines and chemokines such as IL-6, IL-1β, and MCP-1, and subsequently initiated intracellular inflammatory in response to myocardial ischemia (Shimamoto et al., 2006). Additionally, in STZ-induced diabetic rats and HG-cultured podocytes, berberine could decrease the secretion of IL-1β, IL-6, and MCP-1, with an alleviated inflammation effect by inhibiting TLR4 expression (Zhu et al., 2018). As mentioned earlier, TLR4 mediated the inflammatory response of MCs regulated by lncRNA under HG condition. Likewise, there was growing evidence showing a key role of TLR4 dysfunction in diabetic podocyte injury. Recently, lncRNA myocardial infarction-associated transcript (MIAT) has been shown to be closely related to diabetic complications (Sun et al., 2018a). For DKD, MIAT targeted and regulated TLR4 expression by acting as a ceRNA of miR-130a-3p, thereby promoting the release of TNF-α, IL-6, and IL-1β and subsequently initiated inflammatory reaction in immortalized podocytes (Zhang et al., 2020b). Conversely, the knockdown of MIAT could apparently decrease the expression of these pro-inflammatory mediators and exerted anti-inflammation effects (Zhang et al., 2020b), which provided potential therapeutic strategies for DKD.

TIMP3 deficiency-medicated MC injury in DKD has been described earlier. In HG-induced podocytes, it was found that the expression of TIMP3 was also decreased when lncRNA 4930556m19rik and lncRNA HOXA cluster antisense RNA2 (HOXA-AS2) were downregulated (Fan and Zhang, 2020; Li and Yu, 2020). Subcellular fraction assay showed 4930556m19rik was mainly located in cytoplasm of podocytes, overexpression of which was able to elevate the levels of TIMP3 and reduce the secretion of IL-1β, TNF-α, and IL-6 by targeting miR-27a-3p (Fan and Zhang, 2020), while HOXA-AS2 with undefined subcellular localization, mainly by sponging miR-302-3p (Li and Yu, 2020).

### Long Non-Coding RNA and Mitochondrial Dysfunction in Podocyte

Mitochondria are the main site for energy metabolism (Spinelli and Haigis, 2018), constantly changing their shape and size through fusion and fission in order to adapt to the intracellular environment, a process called mitochondrial dynamics. Aberrant mitochondrial fission is a key step to increase ROS production and would lead to podocyte injury (Gujarati et al., 2020), which has been demonstrated to be largely regulated by dynein-associated protein 1 (DRP1), a GTPase that play a crucial role in mitochondrial fission (Chang and Blackstone, 2010). Instead, inhibition of DRP1 significantly decreased the level of mitochondrial ROS, rectified podocyte PFs effacement, and reduced proteinuria in diabetic mice (Ayanga et al., 2016). Recently, the role of lncRNA in HG-induced excessive mitochondrial fission in podocytes has attracted attention. Deng et al. (2020) reported that lncRNA MEG3 was overexpressed in HG-stimulated podocyte cytoplasm, with increased DRP1 expression, upregulated phosphorylation levels, and promoted DRP1 translocation from cytoplasm to mitochondria, causing excessive mitochondrial fission and resulting in podocyte injury, which provided an experimental basis for finding the etiology of DKD progression.

### Long Non-Coding RNA and Autophagy in Podocyte

It had been reported that autophagy activation was negatively correlated with podocyte injury in a mouse model of lupus nephritis (Zhou et al., 2019). Diabetic mice with deficient podocyte autophagy showed decreased podocyte density, thickening of GBM, and disappearance of FPs (Lenoir et al., 2015), suggesting the significance of appropriate maintenance of autophagy activation of podocytes. In recent years, lncRNA has been shown to be a negligible player in dysregulation of podocyte autophagy. LncRNA SOX2OT, acting as a sponge of miR-9, could promote the expression of SIRT1 and resulted in an increased expression of autophagy-related proteins such as Beclin-1, LC3-II, and Atg7, and induced elevated levels of autophagy, which mitigated podocyte injury under HG conditions (Zhang et al., 2019c). SIRT1 was a nicotinamide adenine dinucleotide-dependent deacetylase and recognized to be renal protective by regulating metabolic homeostasis, autophagy, inhibiting inflammation *etc*. (Wang et al., 2019c). SIRT1 knockdown plus HG treatment significantly inhibited the expression of podocyte markers including ZO-1, P-cadherin, and nephrin (Dong et al., 2021b). Moreover, it was shown that lncRNA sperm-associated antigen 5 antisense RNA1 (SPAG5-AS1), primarily expressed in the cytoplasm of HG-induced podocytes, was able to increase the expression of SPAG5 by interacting with ubiquitin specific peptidase 14 (USP-14) and subsequently activated AKT/mTOR signal transduction, with the result that inhibition of podocyte autophagy induced by HG, silencing of SPAG5-AS1 inhibited the expression of caspase-3, caspase-9, and Bax, and reversed the low expression of bcl-2 (Xu et al., 2020).

Additionally, Feng et al. (2018) found lncRNA upregulated expression of GM5524 but downregulated expression of GM15645 in kidney tissues in a mouse model of DN, which were also observed in HG-stimulated mouse podocytes, along with increased expression levels of autophagy-related protein such as Atg5, Atg7, LC3-II, and LC3-II/LC3-I, and increased autophagosomes, while the results were reversed by GM5524 downregulation or GM15645 upregulation. It would be of interest to further determine the mechanisms through which these lncRNAs affected podocyte autophagy signaling pathway, through directly modulating autophagy at transcriptional or posttranscriptional levels, or *via* some other pathway cross talk.

### Long Non-Coding RNA and Apoptosis in Podocyte

Apoptosis is one of the main causes to accelerate podocyte density reduction (Shankland, 2006). Once residual podocytes were unable to completely cover GBM, the integrity of GFB would be destructed and the development of proteinuria might speed up and accelerate glomerulosclerosis (Li et al., 2007). Of particular interest was that lncRNA PVT1, which was previously overexpressed in HG-induced MCs and led to hyperproliferation in a miR-23b-3p/NF-κB manner (Zhong et al., 2020), was also highly expressed in podocyte nuclei under HG conditions, and its abnormal expression was related to reduced methylation levels of the CpG island of forkhead box A1 (FoxA1) and thereby inhibited FoxA1 expression (Liu et al., 2019), the latter of which was a DNA binding protein of the forkhead family and involved in metabolic regulation (He et al., 2017). Notably, silencing of PVT1 or overexpression of FOXA1 inhibited Bax and caspase-3 expressions and promoted Bcl-2 expression, and further prohibited the apoptosis and injury of podocytes in DN mice along with improvement of clinical indexes including creatinine and BUN (Liu et al., 2019).

In addition, high levels of lncRNA small nucleolar RNA host gene 16 (SNHG16) was found in serum of patients with DN and in podocytes treated with HG (He and Zeng, 2020). The overexpression of SNHG16 broke the balance of antiapoptotic protein (Bcl-2) and pro-apoptotic proteins (Bax and caspase-3) by targeting miR-106a, and finally resulted in apoptosis in podocytes (Li et al., 2020b).

Further studies showed that lncRNA CDKN2B-AS1 might also play an indispensable role in HG-induced podocyte apoptosis, in addition to its participation in MC inflammation (Li et al., 2020b). It was found that the overexpression of lncRNA CDKN2B-AS1 decreased protein levels of Bcl-2 but increased levels of Bax, together with the upregulated expression of TGF-β1, FN, and col-I. In contrast, silencing of CDKN2B-AS1 triggered reversed expression of these two apoptosis-related proteins and prohibited expression of the fibrotic proteins (Xiao et al., 2021). The researchers implicated that CDKN2B-AS1 could putatively act as a sponge of miR-98-5p and increase the levels of its target protein notch homolog 2 (NOTCH2), which was an important regulator of renal fibrosis (Huang et al., 2018; Xiao et al., 2021).

## Long Non-Coding RNA and Tubular Epithelial Cell Injury

Tubular epithelial cells (TECs), the main cells of renal tubular interstitium, hold powerful metabolic activity and potential ability of proliferation and secretion (Liu et al., 2018). Tubular atrophy and inflammatory cell infiltration could be observed in the early stage of DKD (Tervaert et al., 2010). Consequently, identifying a more sensitive index and timely intervention for TEC injure possess considerable significance to delay the progress of DKD.

### Long Non-Coding RNA and Inflammation in Tubular Epithelial Cell

Prior studies found that pro-inflammatory cytokines such as IL-1 and TNF-α involved in glomerular injury could simultaneously stimulate proximal renal tubular cells to synthesize TGF-β1 (Phillips et al., 1996), which induced interstitial fibrosis and led to DKD progression. Recently, Feng et al. (2019) found that brown fat lncRNA1 (Blnc1) was highly expressed in the serum of DN patients. In HK-2 cells cultured in HG, inhibition of Blnc1 expression could remarkably decrease the expression of TNF-α, IL-6, and IL-1β and fibrosis-related proteins (PTEN, FN, col-I, and col-IV) through upregulating the nuclear factor erythroid 2-related factor 2 (NRF2)/heme oxygenase-1 (HO-1) signaling pathway. The NRF2/HO-1 signaling pathway has been demonstrated in antioxidant reaction in osteoarthritis *via* inhibiting NLRP3 inflammasome (Chen et al., 2019c).

Dramatically, lncRNA 9884 was a novel SMAD3-dependent non-coding RNA, highly expressed in the nuclei of TECs and MCs of db/db mice, which could directly modulate the expression of MCP-1 at transcriptional level and promoted the infiltration of leukocytes (Zhang et al., 2019d), the latter of which has also been observed in diabetic models and clinical samples of DKD (Galkina and Ley, 2006; Yang and Mou, 2017). Silencing of lncRNA 9884 significantly decreased the expression of MCP-1, TNF-α, and IL-1β, with reduced glomerular matrix deposition, improvement of glomerulosclerosis, and alleviated microalbuminuria excretion (Zhang et al., 2019d), indicating a pivotal role of lncRNA in dysfunction of tubular cells in the context of diabetes.

### Long Non-Coding RNA and Pyroptosis in Tubular Epithelial Cell

Pyroptosis means as a pro-inflammatory programmed cell death, characteristic by activation of the caspase family and mediated by gasdermin D (GSDMD), generally without membrane destruction and accompanied by the participation of neutrophils to clear intracellular necrosis material, which is different from apoptosis manifested as the form of rupture of the cell membrane and the participation of macrophages (Kovacs and Miao, 2017; Shi et al., 2017). New discovery reported NLRP3 inflammasome was an important initiator of pyroptosis by promoting the activation of caspase-1 and accelerating the cleavage of GSDMD (Platnich and Muruve, 2019). Recently, an *in vitro* experiment conducted by Xie et al. (2019) showed that the overexpression of lncRNA growth arrest-specific transcript 5 (GAS5) reduced the secretion of pro-inflammatory cytokines (TNF-α, IL-6, and MCP-1) and inhibited the expression of NLRP3, caspase-1, and GSDMD-N by downregulating miR-452-5p levels, with alleviated oxidative stress responses and pyroptosis in HG-induced TECs. Similar results were illustrated in HG-induced HK-2 cells where KCNQ1OT1 expression was increased (Zhu et al., 2020). It was found that inhibition of KCNQ1OT1 decreased the production of pro-inflammatory cytokines (TNF-α, IL-6, and MCP-1) and inhibited the expression of NLRP3, caspase-1, and GSDMD-N triggered by HG stimulation *via* upregulating the expression levels of miR-506-3p (Zhu et al., 2020). Unfortunately, to date, the involvement of lncRNAs in the regulation of pyroptosis in TECs mainly implemented at cell levels under HG conditions, and further diabetic animal models or clinical experiments are in need.

### Long Non-Coding RNA and Epithelial Mesenchymal Transformation in Tubular Epithelial Cell

Epithelial mesenchymal transformation (EMT) usually refers to a biological process of epithelial cells transforming into cells with mesenchymal phenotype under stimulating factors and promotes the progression of fibrosis, characterized by downregulation of epithelial cadherin (E-cadherin) and upregulation of vimentin and neural cadherin (N-cadherin), and driven by numerous transcription factors including SNAILl, TWIST, and zinc-finger E-box binding (ZEB) (Lamouille et al., 2014). EndMT occurring in glomeruli is one of the important mechanisms of diabetic renal fibrosis and closely related to phenotypic transformation of TECs as mentioned earlier, jointly promoted the progress of DKD. Interestingly, lncRNA MALAT1 was shown to boost the process of EMT of lens epithelial cells in addition to its participation in endothelial inflammatory responses (Puthanveetil et al., 2015; Ye et al., 2020). It was demonstrated that activation of the Wnt/β-catenin signaling pathway significantly decreased the expression of epithelial marker E-cadherin but increased the expression of interstitial marker α-SMA, accompanied by the overexpression of lncRNA MALAT1 in HG-induced HK-2 cells (Zhang et al., 2019a). Another interesting case involved in EMT of TECs was that lncRNA OIP5-AS1 could directly target miR-30c-5p and regulate the expression of E-cadherin and N-cadherin to participate in EMT and renal fibrosis in DN mice and HG-cultured HK-2 cells (Fu et al., 2020).

In contrast, the levels of lncRNA ZEB homeobox 1 antisense 1 (ZEB1-AS1) were markedly lower in kidney tissues of DN patients in comparison with healthy controls. Decreased expression of ZEB1-AS1 was also observed in HK-2 cells treated with HG compared with normal glucose stimulation, with simultaneous elevation of mesenchymal markers α-SMA and vimentin and diminished expression of epithelial marker E-cadherin (Meng et al., 2020). The overexpression of ZEB1-AS1 significantly upregulated BMP7 levels, partially restored the expression of E-cadherin, and decreased α-SMA and vimentin expression resulted from HG stimulation *via* its interaction with miR-216a-5p, which further inhibited EMT of HK-2 cells and renal fibrosis (Meng et al., 2020).

### Long Non-Coding RNA and Apoptosis in Tubular Epithelial Cell

Concurrent with other renal resident cells, apoptosis mediated by lncRNAs also occurred in TECs. In a rat model of DN, it was observed that lncRNA urothelial carcinoma associated 1 (UCA1) was downregulated in the renal cortex (132). *In vitro*, the researches indicated the levels of UCA1 promoted in HG-stimulated HK-2 cells (132). The overexpression of UCA1 could inhibit the synthesis of caspase-1, IL-1β, and NLRP3 *via* directly targeting miR-206 and exerted the renal protective effect (Yu et al., 2022). Another experiment was carried out by Wang et al. (2019b) that the expression of lncRNA 00462 increased in renal tissue of DN patients and HG-induced HK-2 cells. Exposure to HG stimulation could significantly cause cell apoptosis manifested as elevation of Bax and reduction of Bcl-2 by inhibiting the AKT signal pathway, while the opposite effect was obtained when 00462 was knocked down.

### Long Non-Coding RNA and Autophagy in Tubular Epithelial Cell

Thioredoxin-interacting protein (TXNIP), a marker for excess unfolded protein response in the endoplasmic reticulum (ER), could activate inflammatory cytokines and induce inflammatory cascade signals activation (Tsubaki et al., 2020; Yang et al., 2020). Under HG conditions, TXNIP expression levels were elevated in HG-cultured HK-2 cells, decrease of which reversed the inhibition of mitochondrial autophagy *via* reducing the phosphorylation levels of mTOR (Huang et al., 2016). Latest research showed that lncRNA NEAT1 could upregulate TXNIP expression *via* acting as a sponge of miR-93-5p and subsequently triggered LPS-cultured HK-2 cell injury, manifested as increased levels of Bax, TNF-α, IL-1β, and IL-6 but decreased levels of Bcl-2 (Yang et al., 2021b). Notably, it has been shown that lncRNA NEAT1 was elevated in MCs after HG stimulation (Huang et al., 2019). It would be interesting to identify whether lncRNA NEAT1 was involved in TXNIP dysfunction-related regulation of mitochondrial autophagy in glomerular tubular cells *in vitro* and *in vivo*.

## Subcellular Localization and Function of Long Non-Coding RNA in Diabetic Kidney Disease

LncRNAs are selectively located in specific subcellular structures in relation to multifarious biological behaviors. Majority of lncRNAs are usually distributed in the nucleus and function as regulators in chromatin organization by recruiting specific regulatory proteins to the chromatin and RNA translating. An interesting case was that lncRNA TCF7 regulated its expression by recruiting the SWI/SNF complex to the promoter of TCF7 to realize the continuous self-renewal of liver tumor cells (Wang et al., 2015). Similar mechanisms were also exemplified in DKD. It was implicated that nuclear lncRNA Dlx6os1 elevated SOX6 expression by recruiting EZH2 to SOX6 promoter in HG-treated SV40 MES13 cells (Chen et al., 2022). LncRNA PVT1 was located in the nuclei of HG-treated podocytes and was implicated to promote the recruitment of H3K27me3 at the FoxA1 promoter region by interacting with EZH2, and thereby inhibiting the expression of FoxA1 (Liu et al., 2019). EZH2 was one of the four core components of PRC2, which was a methyltransferase and recruited to specific genomic locations where it trimethylated H3K27 to repress the transcription of specific genes (Schuettengruber et al., 2007; Ku et al., 2008; Khalil et al., 2009; Laugesen et al., 2019; Duan et al., 2020). These studies suggested that epigenetic mechanisms that regulate histone methylation with dysregulation of lncRNAs might be critically involved in the development of DKD. Additionally, lncRNA 9884 could directly regulate the expression of MCP-1 at the transcriptional levels to participate in inflammatory injury of TECs in DKD (Zhang et al., 2019d), indicating its function as a potential transcriptional regulator. Interestingly, we previously showed that in podocytes exposed to HG stimulation, lncRNA MALAT1 was involved in podocyte injury through activating β-catenin nuclear translocation with SRSF1 as an important mediator, the former of which in turn regulated MALAT1 transcription *via* binding to its promoter region (Hu et al., 2017). Such nuclear location of lncRNA showed critical roles in cell malfunction under pathological conditions *via* its interplay with nuclear regulatory factors that could move freely between cytoplasm and nucleus. Nevertheless, data were still limited relating to nuclear localization of these lncRNAs and their unique functions in intrinsic renal cells and pathogenesis of DKD.

In addition to its nuclear distribution, some other lncRNAs are transported to the cytoplasm and got involved in the expression of target genes by interacting with certain RNA molecules or proteins. Cytoplasmic lncRNAs acting as a bait for miRNA and subsequently regulated the expression of target genes seemed to be another critical and efficient way to exercise their functions (Poliseno et al., 2010), and usually was known as the “sponge effect.” For example, MEG3-targeted miR-181a (Zha et al., 2019), CASC2-targeted miR-135a-5p (Zhu et al., 2021), HCP5-targeted miR-93p-5p (Wang et al., 2021b), 1700020I14Rik-targeted miR-34a-5p (Li et al., 2018), RMRP-targeted miR-1a-3p (Yang et al., 2021a), H2K2-targeted miR-44a/b (Chen et al., 2019b), MIAT-targeted miR-130a-3p (Zhang et al., 2020b), ZEB1-AS1-targeted miR-216-5p (Meng et al., 2020), and NR_038323-targeted miR-324-3p (Ge et al., 2019) were implicated in HG-triggered injury of resident cells including MCs, tubular cells, and podocytes, and in the pathogenesis of DKD. Cytoplasmic lncRNAs could also initiate activation of some downstream signaling pathways by directly binding to intermediate proteins. It has been indicated that cytoplasmic lncRNA MUF promoted liver tumorigenesis by interacting with annexin A2 protein and miR-34a (Yan et al., 2017). LncRNA RPPH1 directly bound with gal-3 to activate the ERK/MEK signaling pathway, which was sufficient to trigger inflammatory responses of MCs under HG conditions (Zhang et al., 2019b). Moreover, lncRNA SPAG5-AS1 mediated HG-induced podocyte autophagy by interacting with USP-14 (Xu et al., 2020).

Of note, the subcellular localization of lncRNAs was not permanent but highly volatile, showing tremendous potentiality in relation to specific functions under specific circumstances. It was found that mitochondrial gene expression could be regulated by lncRNAs that clustered in mitochondria. LncRNA RMRP, located in nucleus, could be exported to cytoplasm *via* CRM1 when binding to Hu antigen R (HuR), and selectively located in the mitochondrial matrix to bind to GRSF1, a mitochondrial protein with relation to oxidative phosphorylation and mitochondrial DNA replication (Noh et al., 2016). Previous study also reported lncRNA RMRP, located in cytoplasm, could participate in the proliferation of HG-cultured MCs by sponging miR-1a-3p (Yang et al., 2021a), whether mitochondrial dysfunction mediated by RMRP was involved in MCs injury, however, needed further exploration. Interestingly, lncRNAs could be actively sorted into exosomes (Gezer et al., 2014) and involved in intercellular transfer (Dong et al., 2016). Some studies have identified a distribution of the cell–cell contact region of lncRNAs, such as lncRNA LASSIE, which was shown to enrich and stabilize endothelial adhesion by interacting with the cell–cell adhesion component PECAM-1 in the cell–cell contact areas (Stanicek et al., 2020). To date, scarce studies have demonstrated further information of the dynamic distribution of lncRNA LASSIE in diabetic endothelial injury or in the development of DKD. Previous studies reported some lncRNAs, located in ribosome, were able to encode small peptides (Huang et al., 2017), and polysomal lncRNAs seemed to have significantly longer 5′ UTR regions, similar to protein-coding transcripts, which might be beneficial to its ribosomal recognition and thus proper subcellular location (Carlevaro-Fita et al., 2016; Huang et al., 2017), and lncRNAs were also detected in the endoplasmic reticulum (ER) (Fazal et al., 2019). Nevertheless, studies on these unique locations of cytoplasmic lncRNAs in the pathogenesis of DKD were rare, leaving their exact biological or pathological functions and molecular mechanisms still mysterious and elusive.

Although the subcellular localization of lncRNAs has recently emerged to be significant in lncRNA biology and pathobiology, related studies in the field of DKD are still limited and failed to arouse enough attention from researches. A majority of the literature on DKD emphasized aberrant alterations of the expression of certain lncRNAs on levels of certain cells, tissues, or organs, with only rough subcellular descriptions confined to cytoplasm or nucleus. Further investigations of this field would be non-negligible and of great interest and significance to uncover the pathogenesis of DKD, which might be driven by the rapid progress in experimental technology and the biology of lncRNAs per se.

## Long Non-Coding RNA as Therapeutic Target for Diabetic Kidney Disease

With an in-depth exploration of lncRNAs, it has been found that targeting some lncRNAs might be of potential therapeutic values for cancer (Wang et al., 2019a). Recently, the clinical significance of lncRNAs for DKD has just begun to get attention. Evidence showed that lncRNA taurine-upregulated gene 1 (TUG1) could regulate the expression of peroxisome proliferator-activated receptor-γ coactivator-1 alpha (PGC-1α) in kidney biopsy tissue of DN patients and HG-cultured podocytes (Shen et al., 2019). PGC-1α is a well-studied cardinal factor regulating biological functions of mitochondria (Puigserver et al., 1998), dysregulation of which has been illustrated to be correlated with abnormal biological function of mitochondria such as increased production of ROS, irregular mitochondrial dynamics, and autophagy disorder (Fontecha-Barriuso et al., 2020). Evidence showed that downregulation of TUG1 contributed to the development of DN by activating ER stress and podocyte apoptosis (Shen et al., 2019). TUG1 overexpression, on the contrary, reduced the expression of extracellular matrix protein such as FN and col-IV, inhibited cell proliferation in STZ-induced diabetic rats and HG-cultured MCs *via* inhibiting the PI3K/AKT pathway (Zang et al., 2019). The aforementioned studies suggested that the overexpression of TUG1 might be beneficial to hinder the progress of DKD and might provide potential therapeutic direction for future research.

Further study showed that a novel lncRNA NR_038323 was dysregulated in HG-cultured HK-2 cells, with concomitant increase in col-I, col-IV, and FN levels, knockdown of which further increased col-I, col-IV, and FN levels whereas the overexpression of which showed a reversal effect (Ge et al., 2019). In renal tissue of DN patients, similar findings were shown that lncRNA NR_038323 was elevated with increased staining of fibrosis markers col-I, col-IV, and FN by immunohistochemistry (Ge et al., 2019). In STZ-induced DN rats, lncRNA NR_038323 seemed to be playing a potential antifibrosis role by targeting the miR-324-3p/DUSP1 axis, as higher levels of lncRNA NR_ 038323 was correlated with attenuated proteinuria to a certain degree and interstitial fibrosis, suggesting a therapeutic potentiality for DKD (Ge et al., 2019). Nevertheless, data on the application of lncRNAs in the treatment of DKD are still in shortage, and further investigations of lncRNAs in the pathogenesis of DKD are in urgent need.

## Conclusion

As one of the most important microvascular complications of diabetes, DKD holds relatively high morbidity and mortality but limited therapeutic options (Forsblom et al., 2011; Thomas et al., 2015; Alicic et al., 2017). Here, we described some abnormally expressed lncRNAs associated with the pathogenesis of DKD (Supplementary Table S1), which participated in a series of physiological and pathophysiological processes of renal inherent cells. As the role of lncRNAs in DKD becomes clearer, potentially novel diagnostic markers, therapeutic targets, and interventions might be possible. Of interest, most of the human genome encodes RNA that does not code for protein (Yelin et al., 2003; van Bakel et al., 2010), which means there might be a considerable number of undefined lncRNAs involved in DKD. Also, it is still not very clear of the interactions among different lncRNAs and their roles in the cell cross talk between resident kidney cells, as cell-to-cell communication has been indicated as an important mechanism in the pathogenesis of DKD (Li et al., 2021b). The dynamics of the subcellular localization of lncRNAs might also exert critical and differential functions under physiological and pathophysiological conditions such as DKD, the data on which are still lacking. Further exploration of this field has the potential to advance our knowledge of the pathogenesis of DKD, as well as diabetes per se, and might contribute to early screening and prevention of the disease.
