# Supplementary material for: Long Non-Coding RNAs in the Pathogenesis of Diabetic Kidney Disease
Source: Front Cell Dev Biol. 2022 Apr 20;10:845371. doi: 10.3389/fcell.2022.845371 (PMC9065414; doi:10.3389/fcell.2022.845371)
Supplement: Supplementary file 1 [file Table1.DOCX]

**Table 1. Selected studies analyzing expression and function of lnRNAs in DKD**

| LncRNA | Differentiated expression | Animal model/cell analyzed | Main subcellular localization | Target | Function | Reference |
| --- | --- | --- | --- | --- | --- | --- |
| MEG3 | upregulated | DN rat  MCs | cytoplasm | miR-181a/EGR-1/TLR4 | inflammation  fibrosis | (Zha et al., 2019) |
| MEG3 | upregulated | MEG3 knockdown mice  podocytes | cytoplasm | DRP1 | excessive mitochondrial fission | (Deng et al., 2020) |
| RPPH1 | upregulated | db/db DN mice  MCs | cytoplasm | galectin-3/MEK/ERK | inflammation  proliferation | (Zhang et al., 2019b) |
| CASC2 | downregulated | MCs | cytoplasm | miR-135a-5p/ TIMP3/JNK | inflammation  proliferation | (Zhu et al., 2021) |
| CASC2 | downregulated | DN patients  MCs | unspecified | miR-133b/ FOXP1 | proliferation  ECM accumulation  oxidative stress | (Zhang et al., 2020c) |
| Gm4419 | upregulated | DN mice  MCs | cytoplasm | NF-κB /NLRP3 | inflammation | (Yi et al., 2017) |
| HCP5 | upregulated | DN patients  MCs | cytoplasm | miR-93-5p/HMGA2/AKT/mTOR | inflammation  proliferation  fibrosis | (Wang et al., 2021b) |
| CTBP1-AS2 | downregulated | DN patients  MCs | unspecified | miR-155-5p/FOXO1 | inflammation  oxidative stress  ECM accumulation  proliferation | (Wang et al., 2020) |
| NNT-AS1 | upregulated | DN patients  MCs | unspecified | miR-214-5p/smad4 | inflammation  ECM accumulation  proliferation | (Geng et al., 2021) |
| NORAD | upregulated | DN patients  MCs | unspecified | miR-485-NRF1 | inflammation  proliferation  fibrosis | (Wang et al., 2021a) |
| NEAT1 | upregulated | diabetic rats  MCs | unspecified | AKT/mTOR | proliferation  fibrosis | (Huang et al., 2019) |
| PVT1 | upregulated | DN patients  MCs | unspecified | miR-23b-3p/NF-κB | proliferation  fibrosis | (Zhong et al., 2020) |
| PVT1 | upregulated | DN patients  DN mice  podocytes | nucleus | FOXA1 | apoptosis | (Liu et al., 2019) |
| Dlx6os1 | upregulated | db/db mice  SV40 MES13 cells | nucleus | SOX6 | proliferation  inflammation  fibrosis | (Chen et al., 2022) |
| CDKN2B-AS1 | upregulated | DN patients  MCs | unspecified | miR-424-5p/ HMGA2 | proliferation  ECM accumulation | (Li et al., 2020b) |
| CDKN2B-AS1 | upregulated | DN patients  podocytes  HK-2 cells | unspecified | miR-98-5p/ NOTCH2 | apoptosis  fibrosis | (Xiao et al., 2021) |
| 1700020I14Rik | downregulated | db/db DN mice  MCs | cytoplasm | miR-34a-5p/Sirt1/HIF-1α | proliferation  fibrosis | (Li et al., 2018) |
| RMRP | upregulated | DN mice  MCs | cytoplasm | miR-1a-3p/JunD | proliferation  fibrosis | (Yang et al., 2021a) |
| H2k2 | upregulated | db/db DN mice  MCs | cytoplasm | miR-449a/b/MEK/ERK | proliferation | (Chen et al., 2019b) |
| NR_033515 | upregulated | DN patients  MCs | unspecified | miR-743b-5p | proliferation  fibrosis  EMT | (Gao et al., 2018) |
| SOX2OT | downregulated | DN mouse  MCs | unspecified | AKT/mTOR | proliferation  fibrosis | (Chen et al., 2021) |
| SOX2OT | downregulated | podocytes | unspecified | miR-9/SIRT1 | autophagy | (Zhang et al., 2019c) |
| MALAT1 | upregulated | diabetic mice  ECs | unspecified | SAA3 | inflammation | (Puthanveetil et al., 2015) |
| MALAT1 | upregulated | HK-2 cells | unspecified | miR-30c/NLRP3 | pyroptosis | (Liu et al., 2020) |
| MALAT1 | upregulated | Diabetic rats  HK-2 cells | unspecified | miR-30c/ELAVL1 | pyroptosis | (Li et al., 2017a) |
| MALAT1 | upregulated | HK-2 cells | unspecified | Wnt/β-catenin | EMT | (Zhang et al., 2019a) |
| KCNQ1OT1 | upregulated | DN patients  MCs  ECs | unspecified | miR-18b-5p/ SORBS2  NF-κB | proliferation  apoptosis  fibrosis | (Jie et al., 2020) |
| KCNQ1OT1 | upregulated | DN patients  MCs | unspecified | miR-18b/HMGA2 | proliferation  oxidative stress  ECM accumulation | (Li et al., 2021a) |
| KCNQ1OT1 | upregulated | DN patients  podocytes | unspecified | miR-23b-3p/Sema3A | inflammation  proliferation  apoptosis | (Fei et al., 2022) |
| KCNQ1OT1 | upregulated | DN patients  HK-2 cells | unspecified | miR-506-3p | oxidative stress  pyroptosis | (Zhu et al., 2020) |
| H19 | upregulated | diabetic mice  ECs | unspecified | miR-29a/TGF-β/SMAD3 | EndMT | (Shi et al., 2020) |
| MIAT | upregulated | podocytes | cytoplasm | miR-130a-3p/ TLR4 | inflammation  apoptosis | (Zhang et al., 2020b) |
| MIAT | upregulated | HK-2 cells | unspecified | miR-182-5p/GPRC5A/ NF-κB | inflammation  apoptosis | (Dong et al., 2021a) |
| 4930556M19Rik | downregulated | podocytes | cytoplasm | miR-27a-3p/TIMP3 | inflammation  apoptosis  fibrosis | (Fan and Zhang, 2020) |
| HOXA-AS2 | downregulated | DN patients  DN rats  podocytes | unspecified | miRNA-302b-3p/TIMP3 | inflammation  proliferation  apoptosis | (Li and Yu, 2020) |
| ANRIL | upregulated | podocytes | unspecified | membrane metallo-endopeptidase | inflammation  oxidative stress  apoptosis | (Cai and Jiang, 2020) |
| ANRIL | upregulated | DN patients  HK-2 cells | unspecified | miR-497/TXNIP | pyroptosis | (Wang and Zhao, 2021) |
| SPAG5-AS1 | upregulated | podocytes | cytoplasm | USP-14/ SPAG5/AKT/mTOR | autophagy  apoptosis | (Xu et al., 2020) |
| GM5524 | upregulated | DN mouse  podocytes | unspecified | caspase-3, Bax, LC3I,  LC3II, Atg5, Atg7, Bcl2 | apoptosis  autophagy | (Feng et al., 2018) |
| GM15645 | downregulated | DN mouse  podocytes | unspecified | caspase-3, Bax, LC3I,  LC3II, Atg5, Atg7, Bcl2 | apoptosis  autophagy | (Feng et al., 2018) |
| Hoxb3os | downregulated | db/db mice  podocytes | unspecified | ATK/mTOR | apoptosis  autophagy | (Jin et al., 2021) |
| SNHG16 | upregulated | DN patients  podocytes | unspecified | miR-106a/KLF9 | oxidative stress  pyroptosis | (He and Zeng, 2020) |
| 00162 | upregulated | DN patients  HK-2 cells  293T cells | unspecified | miR-383/HDAC9 | apoptosis | (Fan et al., 2020) |
| MEG8 | upregulated | DN patients  podocytes | unspecified | miR-770-5p | apoptosis | (Zhang et al., 2020a) |
| Blnc1 | upregulated | DN patients  DN rats  HK-2 cells | unspecified | NRF2/HO-1  NF-κB | fibrosis  inflammation  oxidative stress | (Feng et al., 2019) |
| 9884 | upregulated | db/db mice  TECs  MCs | nucleus | MCP-1 | inflammation | (Zhang et al., 2019d) |
| GAS5 | downregulated | HK-2 cells | unspecified | miR-452-5p/ NLRP3//GSDMD | oxidative stress  pyroptosis | (Xie et al., 2019) |
| NEAT2 | upregulated | HK-2 cells | unspecified | miR-206 | pyroptosis  infalmmation | (El-Lateef et al., 2022) |
| UCA1 | downregulated | DN rats  HK-2 cells | unspecified | miR-206 | apoptosis  inflammation | (Yu et al., 2022) |
| OIP5-AS1 | upregulated | db/db mice  HK-2 cells | unspecified | miR-30c-5p | EMT | (Fu et al., 2020) |
| ZEB1-AS1 | downregulated | DN patients  HK-2 cells | cytoplasm | miR-216a-5p/BMP7 | EMT | (Meng et al., 2020) |
| 00462 | upregulated | DN patients  HK-2 cells  HKC cells | unspecified | AKT | apoptosis | (Wang et al., 2019b) |
| TUG1 | downregulated | DN patients  podocytes | unspecified | PGC-1α | apoptosis | (Shen et al., 2019) |
| TUG1 | downregulated | diabetic rat  MCs | unspecified | PI3K/AKT | proliferation  fibrosis | (Zang et al., 2019) |
| NR_038323 | upregulated | DN patients  DN rats  HK-2 cells | cytoplasm | miR-324-3p/DUSP1/ p38MAPK/ERK1/2 | fibrosis | (Ge et al., 2019) |
